# Preliminary experience with 3T magnetic resonance elastography imaging of the liver

**DOI:** 10.4102/sajr.v25i1.2072

**Published:** 2021-05-12

**Authors:** Anagha Joshi, Mridula M. Muthe, Vikrant Firke, Harshal Badgujar

**Affiliations:** 1 Department of Radiology, Lokmanya Tilak Municipal Medical College, Lokmanya Tilak Municipal General Hospital, Mumbai, India

**Keywords:** magnetic resonance elastography, liver fibrosis, 3T, cirrhosis, stiffness, liver stiffness measurement, chronic liver disease, non-alcoholic steatohepatitis

## Abstract

**Background:**

Magnetic resonance elastography (MRE) is a promising non-invasive technique for the identification and quantification of hepatic fibrosis. This manuscript describes our early experience with MRE for the assessment of the presence and staging of liver fibrosis on a 3T magnetic resonance imaging (MRI) system.

**Objectives:**

The purpose of this study was to describe the MRE physics, procedure, interpretation and drawbacks, along with a few recommendations as per our experience.

**Method:**

Magnetic resonance elastography was performed on 85 patients with a 3T MRI and the images were analysed both qualitatively and quantitatively. Liver stiffness was assessed by drawing freehand geographic regions of interest on the elastograms to cover the maximum portion of the hepatic parenchyma within the 95% confidence maps on each slice. Correlation with histopathology was performed whenever available.

**Results:**

Of the 80 patients who met the inclusion criteria, 41 patients displayed a normal liver stiffness measurement (LSM) and 39 patients had a raised LSM. In the patients who had a raised LSM, 14 patients had Stage I–II fibrosis, 8 patients had Stage II–III fibrosis, 6 patients had Stage III–IV fibrosis, 4 patients had Stage IV fibrosis or cirrhosis and 7 patients had non-alcoholic steatohepatitis. The mean thickness of the waves increased with increasing stages of fibrosis. The waves became gradually darker medially in patients with normal LSM as compared to the patients with raised LSM. Histopathology with METAVIR scoring was available in 46 patients, which agreed with the MRE findings in all except two patients.

**Conclusion:**

Magnetic resonance elastography is a suitable non-invasive modality for the identification and quantification of hepatic fibrosis.

## Introduction

Chronic liver diseases (CLDs) are a major cause of morbidity and mortality in the world. Hepatic fibrosis is the result of different CLDs caused by viral infections like hepatitis B (HBV) and hepatitis C (HCV), alcohol abuse, non-alcoholic fatty liver disease (NAFLD), autoimmune disease and metabolic disorders. Alcoholic liver disease (ALD) is the commonest cause of cirrhosis in India.^1^ The development of fibrosis is proven to be the strongest predictor of prognosis in patients with NAFLD.^2^ Abnormal fibrinogenesis in response to chronic liver injury results in hepatic fibrosis, which, in advanced stages, further leads to cirrhosis.

Hepatic fibrosis is a dynamic process that can be reversed with effective treatment if detected early. Untreated, it can progress to cirrhosis causing hepatic failure and its complications like variceal bleeding, ascites, portal hypertension and hepatocellular carcinoma.^3,4,5^ Therefore, the early detection of the development and extent of liver fibrosis and recognition of cirrhosis is critical for determining the prognosis and appropriate clinical management of CLD.^6,7^ Liver fibrosis is a predictor of liver function and preoperative assessment of fibrosis is an important predictor of the risk of liver failure after surgery.^8^

Liver biopsy is the current gold standard for the detection and staging of fibrosis.^1,4,8,9^ However, it is an invasive method, has poor patient acceptance, evaluates a small volume of liver parenchyma and can cause undesired complications, such as bleeding and mortality in some instances.^1,8,10^ Sampling variability resulting from heterogeneity in the liver parenchyma and interobserver variability in the assessment of the histopathological specimens also add to its limitations.^11,12^

During the last several years, new non-invasive methods of assessing liver fibrosis have been developed and investigated, including serum tests, diffusion-weighted imaging, ultrasound-based transient elastography and magnetic resonance elastography (MRE).^7,8^ Of these, MRE is the most accurate method to date for detecting and staging liver fibrosis, especially in the early stages of fibrosis.^13,14^ It is also useful in the follow-up staging evaluation and for the assessment of response to treatment.

This article describes our early experience with MRE for the assessment of the presence and staging of liver fibrosis at 3T magnetic resonance imaging (MRI). It also ellaborates on the related physics, procedure, interpretation and drawbacks, along with a few recommendations.

## Methods

This was a retrospective, observational study performed at the Lokmanya Tilak Municipal General Hospital spanning from 20 June 2020 to 20 November 2020. All patients with suspected liver disease referred for the presence and quantification of liver fibrosis were included. Magnetic resonance elastography examinations were performed on 85 patients using a 3.0T whole-body MR imager (Philips Ingenia) with a phased-array torso coil. Excluded patients were those with hepatic iron overload, acute hepatitis and congestive cardiac failure, patients with a history of claustrophobia and patients with contraindications for MRI, such as the presence of a pacemaker or cochlear impants. Four patients were excluded from the study because of poor breath-hold, leading to poor image quality and a region of interest (ROI) of less than 700 pixels. One patient was excluded because of significant iron overload with T2* values of 6.32 ms.

Of the 80 patients who underwent MRE, 26 patients were known to have HBV infection, ALD was suspected in 30 patients, 8 patients were known cases of HCV, 15 patients were suspected cases of non-alcoholic steatohepatitis and 1 patient was a case of Bechet’s disease on treatment with methotrexate.

### Technique

Magnetic resonance elastography was performed on a Philips Ingenia 3.0T whole-body MR scanner. All examinations were performed after the patient fasted for at least 6 h. Because MRE is a breath-hold sequence, it was acquired with the patient holding breath, preferably in end expiration over 18 s.

Four axial slices, each 10 mm thick, were placed in the upper middle part (widest part) of the liver to obtain the largest cross-section of liver parenchyma for liver stiffness measurement (LSM). The MRE sequence in our 3.0T MRI system (Philips Ingenia) is a modified gradient recalled echo (GRE) sequence with the following parameters: repetition time/echo time (TE), 50/21.1 ms; flip angle, 30°; bandwidth, 250 Hz/pixel; field of view, 450 × 403 and matrix, 300 × 86.

The scan was commenced with the acquisition of a T2-weighted sequence in the axial plane to assess the morphology, signal intensity characteristics of the liver parenchyma, presence of focal lesions, if any, and associated findings. If diffuse hypointensity of the liver was observed on T2-weighted images (T2WI), an iron storage disorder was suspected and a T2* sequence was added to the protocol to assess for iron overload. The study was aborted in the case of hepatic iron overload to avoid spurious results. In-phase and out-of-phase images were acquired to detect fatty infiltration of the liver, after which an MRE sequence was acquired. In the case of focal hepatic lesions identified on T2WI, the study was further modified to assess these lesions in detail including acquisition of post-contrast imaging.

Magnetic resonance elastography acquisition was processed to generate quantitative maps displaying the stiffness of the tissue. The acquired MRE data were processed automatically using software with an inversion algorithm to generate greyscale and colour elastograms with superimposed confidence maps.

### Image analysis

All studies were evaluated independently by a radiologist with 30 years of experience and two radiologists with 7 years of experience as well as a senior resident. Correlation with histopathology was performed when available, with the pathologist blinded to the MRE results.

Initially, the quality of the MRE images obtained was assessed to confirm proper technique and reportability. The application and transmission of mechanical waves were verified during the scanning itself. The application of mechanical waves by the passive driver through the liver was confirmed by the presence of a signal void in the subcutaneous region on the magnitude images at the site of the passive driver placement. The transmission of mechanical waves through the hepatic parenchyma was confirmed by the presence of alternate black and grey waves seen on the phase images. In the case of failure to visualise these waves, the connection between the plastic tube connecting the active and passive drivers and the status of the active driver were checked. Furthermore, the wave images were checked to look for adequate propagation of the waves parallel to the hepatic surface with adequate amplitude, having a good signal-to-noise ratio. Finally, the elastogram images with superimposed 95% confidence maps were studied to validate the diagnostic quality.

Both qualitative and quantitative evaluation of the MRE images was performed. For qualitative assessment, the wave images were studied for the thickness of each wave and the brightness. The colour elastograms were reviewed on a scale of 0 kPa – 8 kPa.

**FIGURE 1:**
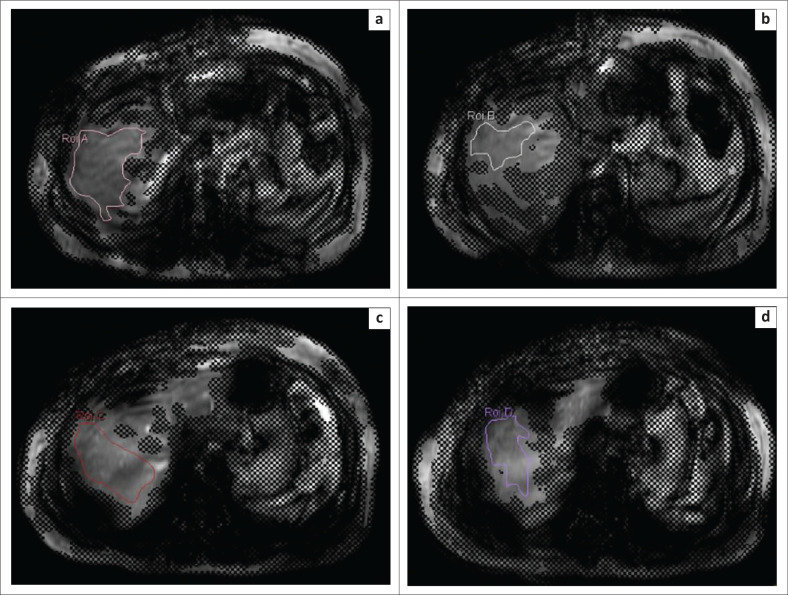
Depiction of the measurement of liver stiffness (a–d), by drawing free-hand regions of interest on all four slices of the magnitude images to cover the maximum hepatic parenchyma not covered by the 95% confidence maps.

Liver stiffness was assessed qualitatively on the MRI console by drawing freehand geographic ROIs on the elastograms to cover the maximum portion of the hepatic parenchyma within the 95% confidence maps on each slice (Figure 1). The ROIs were placed 1 cm away from and parallel to the liver margin. Large vessels, fissures, gall bladder fossa and any areas affected by cardiac and vascular artefacts were excluded. Cross-hatched areas of 95% confidence map overlay and artefactual areas were avoided. Regions of interest were drawn on magnitude images with confidence map overlay. The mean of all the values obtained from four different slices, which were automatically calculated using the in-built software, were reported. The stage of fibrosis was interpreted in accordance with the guidelines suggested by SK Venkatesh et al.^15^ (Table 1). The presence or absence of fibrosis was correlated with liver histopathology whenever available.

**TABLE 1:** Interpretation of the stage of fibrosis based on the liver stiffness measurement as suggested by Venkatesh et al.

LSM	Interpretation
< 2.5 kPa	Normal
2.5 – 2.9 kPa	Normal or Inflammation
2.9 – 3.5 kPa	Stage 1-2 Fibrosis
3.5 – 4 kPa	Stage 2 - 3 Fibrosis
4 – 5 kPa	Stage 4 - 5 Fibrosis
> 5 kPa	Stage 5 Fibrosis or Cirrhosis

*Source*: Venkatesh SK, Yin M, Ehman RL. Magnetic resonance elastography of liver: Technique, analysis, and clinical applications. J Magn Reson Imag. 2013;37(3): 544–555. https://doi.org/10.1002/jmri.23731

LSM, liver stiffness measurement.

## Ethical considerations

This retrospective study was performed with de-identified patient data. The Institutional Ethics Committee Human Research at the Lokmanya Tilak Municipal Medical College and General Hospital granted ethics approval for this study (reference D020190117). Written informed consent was obtained from all patients before MRE.

## Results

**FIGURE 2:**
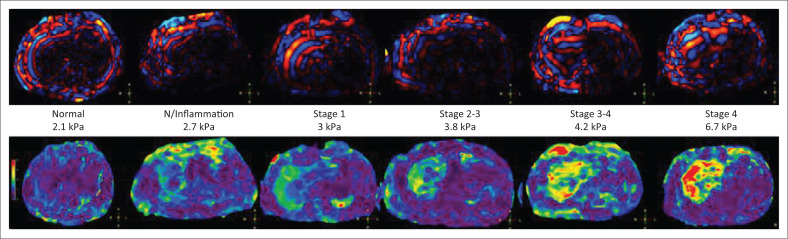
Wave and colour images of patients ranging from normal liver stiffness measurement to stage 4 fibrosis.

**FIGURE 3:**
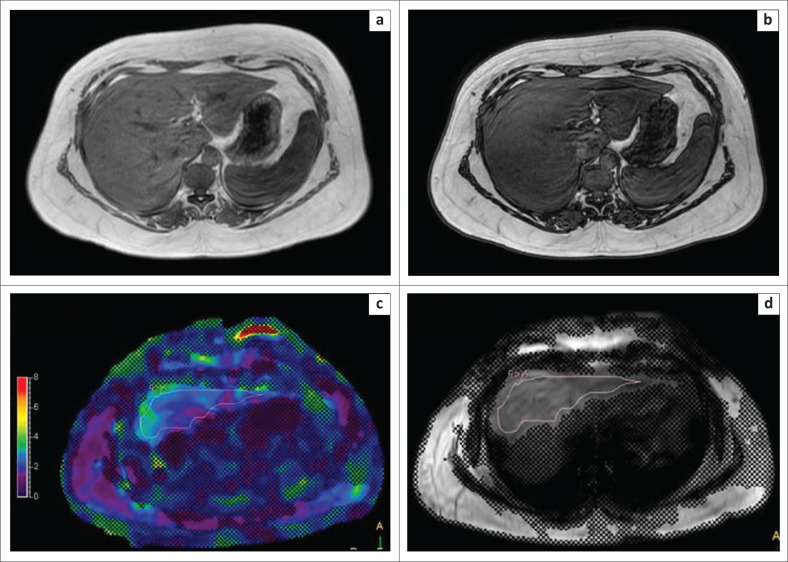
Non-alcoholic steatohepatitis. In-phase (a) and out-of-phase (b) images reveal a drop in signal on the out-of-phase image, indicating the presence of elevated hepatic fat. The colour (c) and magnitude (d) images reveal a liver stiffness measurement of 2.78 kPa.

The final study population included 80 patients, 34 females and 51 males, with ages ranging from 17 to 67 years. A normal LSM was recorded in 41 of the 80 patients who underwent liver MRE. Of the 39 patients who had raised LSMs, 14 patients had Stage I–II fibrosis, 8 patients had Stage II–III fibrosis, 6 patients had Stage III–IV fibrosis, 4 patients had Stage IV fibrosis or cirrhosis (Figure 2) and 7 patients had hepatic steatosis with LSM values between 2.5 and 2.9, which indicated either normal stiffness or inflammation (Figure 3). Sixty-eight patients had fatty infiltration of liver on in-phase and out-of-phase images.

**FIGURE 4:**
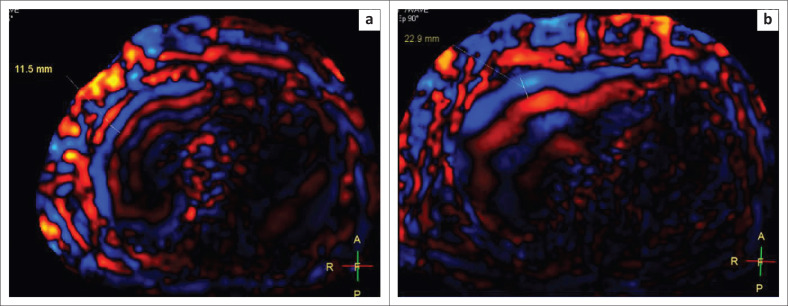
Wave images from a patient with normal liver stiffness measurement (a) and raised liver stiffness measurement (b) show an increase in the thickness of the waves (11.5 mm – 22.9 mm) as the liver stiffness measurement increases.

**FIGURE 5:**
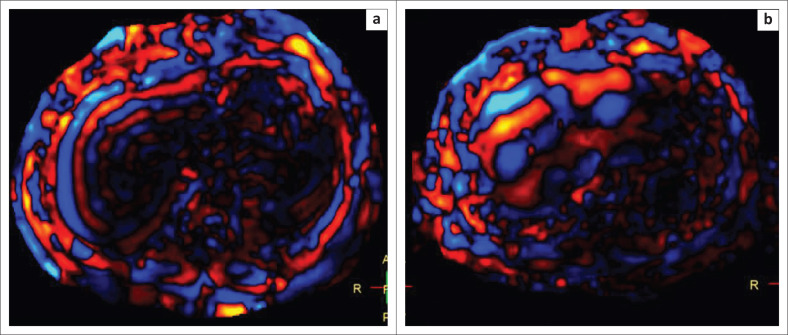
Wave images of a patient with a normal liver stiffness measurement (a) and raised liver stiffness measurement (b) show darker medial waves in the patient with a normal liver stiffness measurement as compared to the patient with a raised liver stiffness measurement.

It was observed that the mean thickness of the waves on the wave images was 10.7 mm in patients with a normal LSM (*n* = 41). The mean wave thickness in patients with Stage I–II fibrosis (*n* = 14) was 11.9 mm and in those with Stage IV fibrosis (*n* = 4), it was 22.25 mm. The thickness of the waves in patients with Stage II–III fibrosis (*n* = 8) was 14.4 mm and in patients with Stage III–IV fibrosis (*n* = 6), it was 15.7 mm. Thus, the thickness of the waves increased with increasing stages of fibrosis (Figure 4). It was also observed that the waves were darker medially in patients with a normal LSM as compared to the patients with a raised LSM. (Figure 5).

Histopathology with METAVIR scoring was available in 46 patients, which agreed with the MRE findings in all except two patients. In both these patients, the fibrosis was overestimated. The histopathology of one of these patients revealed ballooning degeneration representing changes of hepatitis and no fibrosis, which could explain the spuriously elevated LSM of 3.3 kPa (Stage I–II fibrosis), whilst in another patient, the histopathology was normal and MRE revealed an LSM of 3.1 kPa (Stage I–II fibrosis).

## Discussion

Magnetic resonance elastography is emerging as the most reliable non-invasive alternative to biopsy in patients with suspected liver fibrosis. It is safe, cost-effective and devoid of sampling errors seen with biopsy. Most patients are fit to undergo MRE of the liver, including those with ascites or obesity.^16^

Magnetic resonance elastography can also be used for monitoring liver fibrosis in such patients and the results of MRE are reproducible.^17,18,19^ The staging of fibrosis with MRE is comparable with biopsy.^20,21,22^ In addition, a few studies have shown that the interobserver variability between different radiologists reading MRE studies is less than that between different pathologists reading histopathology specimens.^23,24^

### Physics

The stiffness of tissues can be characterised using MRE non-invasively. In this technique, mechanical waves are applied to the tissues, producing small displacements in the tissue. These displacements, which occur in the horizontal plane, are called ‘shear waves’. The difference in the wavelengths of shear waves propagated through tissue depends on the stiffness of the tissue and is used to produce liver stiffness maps. Low-frequency shear waves in the range of 20 Hz – 200 Hz can be used because they undergo less attenuation and their wavelengths are in a measurable range in tissue.^25,26^ Typically, a 60-Hz shear wave frequency is used across vendors as the low frequency of these waves aids in better tissue transmission as compared to high-frequency waves and is also comfortable for the patients.^6,15,27^

Apart from the frequency, the amplitude of the passive driver is also important as it determines the intensity of the vibrations produced on the anterior abdominal wall.^28^

### Procedure

The technique can be easily implemented on a 1.5T or 3T MR system with additional hardware to generate the mechanical shear waves and software for processing the elastogram maps. The LSM depends on the frequency used, and hence, the LSM is the same with 1.5T or 3.0T as long as the same frequency is used for MRE.^15,29^

Magnetic resonance elastography involves three major steps: (1) use of an external driver (vibration source) to generate mechanical waves in tissue, (2) imaging the waves with a special MRI sequence that is sensitive to motion and (3) processing the resultant data using an inversion algorithm to generate quantitative maps displaying the stiffness of tissue.

The active driver, which produces a continuous acoustic vibration of 60 Hz, is placed outside the scanner room. A 25-foot-long plastic tube connects the active driver to the passive driver, which is in close contact with the body and through which the acoustic vibrations are transmitted to the liver. The passive driver is an MR-compatible plastic disc, 19 cm in diameter. It has a flexible membrane that transmits the vibrations into the body, inducing the mechanical shear waves.

The sequences that can be used for MRE are GRE, spin echo, balanced steady-state free precession and echo-planar imaging.^30^ These sequences are used to track the shear waves traversing through the hepatic parenchyma.^27^ Various MRI vendors have different parameters for MRE sequences, which have been outlined in detail by the Radiological Society of North America Quantitative Imaging Biomarkers Alliance.^28^

The continuous mechanical shear waves transmitted in the tissues result in tissue displacement, which induces cyclical motion of the spins, generating a measurable phase shift in the presence of motion-encoded gradients.^25^ Wave images at different time points are obtained by adjusting the phase offset between the mechanical motion and the oscillating motion-encoded gradients. A magnitude and phase image is produced for each phase offset.^15,30^ Four phase offsets evenly placed over one cycle of motion are used in the 2D-GRE MRE sequence.

Magnitude images provide anatomic information and phase images provide wave motion information.^28,30^ These magnitude and phase images are raw data images. An inversion algorithm is applied to these images, which measure the magnitude of the complex shear modulus.^6^ Elastograms or stiffness maps, which include a colour wave image, a greyscale elastogram and a colour elastogram image, are generated automatically by the scanner computer within 2 min of the MRE sequence. The colour wave image depicts the propagation of the mechanical shear waves through the liver parenchyma. The greyscale elastogram is used for quantitative assessment, whereas the colour elastogram is used for qualitative assessment. The correlation coefficient of polynomial fits is used with the inversion algorithm to generate 95% confidence maps, which differentiate between accurate and less accurate LSM resulting from low-wave amplitude using a ‘checker board’ overlay.^28,31^

Greyscale and colour elastograms with and without superimposed confidence maps are generated. The LSM is depicted in kPa units on greyscale images. The colour elastogram maps depict the stiffness in the range of 0 kPa – 8 kPa.

Magnetic resonance elastography is performed with the patients in a state of at least 4–6 h of fasting.^31^ Fasting is important because, after eating food, the portal blood supply to the liver increases, which is compensated for by autoregulation of sinusoidal resistance to maintain normal portal pressure; however, in the presence of fibrosis, autoregulation is faulty, leading to postprandial elevation of portal pressure, which increases liver stiffness.^15,32,33^ Similarly, in patients undergoing repeat follow-up MRE examinations, fasting is essential to ensure optimum comparison.^28^

### Interpretation

**FIGURE 6:**
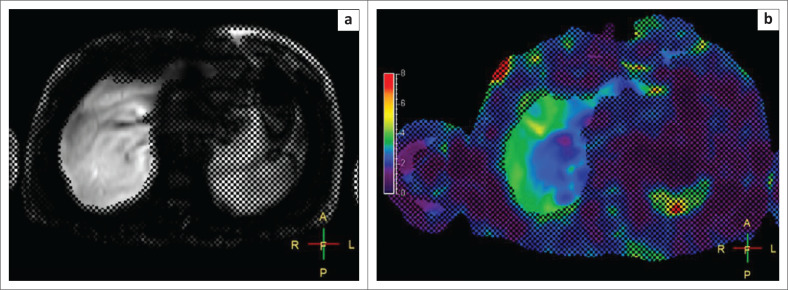
Magnitude (a) and colour images (b) reveal an optimum study with the maximum part of the liver not covered by the 95% confidence map.

**FIGURE 7:**
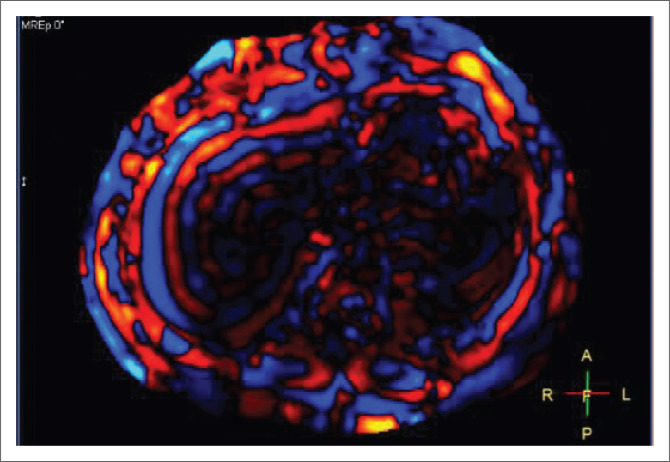
Good quality elastogram with regular waves formed parallel to the liver surface.

The interpretation should first begin with assessing the quality of the elastogram. In a good study, the maximum part of the liver is not covered by 95% confidence maps (Figure 6), and on wave images, the waves are formed parallel to the outer surface of the liver and each other (Figure 7). In a poor-quality study, the waves are of low amplitude (dark) and irregular or chaotic in pattern. In a non-diagnostic study, the maximum part of the liver is covered by the 95% confidence maps.

The thickness of the waves on colour wave images continues to increase in patients with progressive grades of fibrosis. In patients with a normal LSM, the waves become darker medially as compared to patients with a raised LSM. This occurs as a result of the increased attenuation of the shear waves by the normal hepatic parenchyma.^28^

The LSM is obtained by drawing geographic ROIs to cover the maximum hepatic parenchyma with superimposed 95% confidence maps on each of the four elastogram slices. Regions of interest should be placed only in regions of the liver that have adequate wave amplitude, avoiding large vessels, fissures, the gall bladder fossa, any areas affected by cardiac and vascular artefacts, areas covered by cross-hatched areas of 95% confidence map overlay and artefactual areas. The mean value obtained from four different levels of the liver along with the stage of fibrosis, if present, is reported.

Depending on the aetiology of the CLD, the degree and extent of fibrosis can vary. Hence, the thresholds used to determine the degree of fibrosis may also vary depending on the aetiology. There have been many research studies with variable cut-off values for different stages of fibrosis in patients with liver parenchymal disease because of various aetiologies. However, most studies have concluded that a cut-off value of 3 kPa is reliable to distinguish patients with normal liver parenchyma from those with fibrosis.^31^

### Drawbacks

Spuriously elevated LSMs are seen in patients with acute hepatitis, biliary obstruction, passive venous congestion, diffuse infiltrative diseases such as sarcoidosis or amyloidosis and neoplastic causes such as lymphoma or multifocal metastasis.^34^ In patients with moderate to severe iron storage disorder, MRE results may be inconclusive because of the low signal-to-noise ratio, especially in GRE-based sequences.^8^ A T2* of 20 ms or lower reflects a higher likelihood of non-diagnostic elastography.^35^

## Recommendations

- As MRE is a breath-hold sequence, it is useful to practise breath hold techniques with the patient before placing the patient on the MR table, especially when performing MRE on scanners with a GRE sequence.
- Optimum monitoring of image quality during the scanning is of utmost importance to avoid repeat studies and non-diagnostic examinations.
- A T2-weighted axial sequence should be incorporated in the MRE protocol, which enables evaluation of the hepatic parenchyma for possible iron overload, apart from the detection of other abnormalities such as focal lesions, biliary dilatation, focal areas of fibrosis related to confluent hepatic fibrosis or healing lesions.
- In the case of suspected iron overload, it is worthwhile to perform a T2* sequence for quantification of iron overload and defer the imaging in the case of moderate to severe iron overload.
- Liver stiffness measurements should always be evaluated in conjunction with clinical and laboratory parameters to avoid errors in reporting, as spuriously raised LSMs are seen in conditions such as acute hepatitis, hepatic congestion, cholestasis secondary to biliary obstruction and hepatic infiltrative disorders.

## Conclusion

Quantification of liver fibrosis is rapidly gaining importance in view of the recent evidence supporting reversal of the early stages of fibrosis by medical therapy. Magnetic resonance elastography has emerged as a reliable non-invasive modality for the assessment of liver fibrosis because of its excellent correlation with histopathology, reproducibility, repeatability, good inter-observer agreement, assessment of a large volume of hepatic parenchyma as opposed to biopsy, the possibility of examination in obese patients and examination of patients with gross ascites. Radiologists need to be aware of this technique, how to assess for and interpret an optimum study and understand the drawbacks of MRE.
